# Rules of Chinese Herbal Intervention of Radiation Pneumonia Based on Network Pharmacology and Data Mining

**DOI:** 10.1155/2022/7313864

**Published:** 2022-04-25

**Authors:** Wenchao Dan, Cong Wu, Chunyan Xue, Jinlei Liu, Xinyuan Guo, Yanjie Lian

**Affiliations:** ^1^Department of Cardiology, China Academy of Chinese Medical Sciences Guang'anmen, Beijing 100053, China; ^2^Graduate School of Beijing University of Chinese Medicine, Beijing 100029, China; ^3^Department of Radiation Therapy, Cancer Hospital, Chinese Academy of Medical Sciences, Beijing 100021, China

## Abstract

**Objective:**

To explore the mechanism and principles of traditional Chinese medicine (TCM) in the management of radiation pneumonia.

**Methods:**

The targets of radiation pneumonia were obtained by screening the GeneCards, OMIM, TTD, DrugBank, and HERB databases, analyzing ADME parameters. In addition, compounds and Chinese herbs that can act on the targets were screened from the TCMSP database. The core target compounds for TCM were used to construct the target-compound, compound-traditional Chinese medicine, and target-compound-traditional Chinese medicine networks. These networks were further used to select the core targets, compounds, and TCM. The binding strength between the core targets and compounds was determined using AutoDock Vina. The trajectory for the molecular dynamics simulation was completed by Desmond version 2020.

**Results:**

A total of 55 active targets in radiation pneumonia were identified. Subsequently, 137 candidate compounds and 469 Chinese herbs were matched. Frequency statistics showed that the Chinese herbs that could interfere with radiation pneumonia were mainly bitter, spicy, and sweet, with both cold and warm properties. Moreover, they mainly belonged to liver and lung channels. The core targets included TNF, IL-6, TGF-*β*1, and TP53. The most important components were quercetin, resveratrol, and (-)-epigallocatechin-3-gallate. Moreover, the most significant traditional Chinese herbs were *Perilla pueraria*, ephedra, *Lonicerae japonicae*, and sea buckthorn. Furthermore, analysis of 222 sets of receptor-ligand docking results suggested that the compounds had good docking activity to their core targets. By combining the docking binding energy, we determined that the chemical compounds had strong binding energy to the targets.

**Conclusion:**

Using network pharmacology, we explored the potential mechanism of TCM in the treatment of radiation pneumonia. The general rules for application of TCM in the treatment of radiation pneumonia were summarized. This study provides baseline information for future research on the development of TCM for the management of radiation pneumonia.

## 1. Background

Radiation pneumonitis (RP), an inflammatory reaction in normal lung tissues, is caused by injuries in the radiation field after radiotherapy for thoracic or other malignant tumors. Clinically, RP is the first stage of radiation-induced lung injury (RILI), a common radiotherapeutic complication in tumor patients. Acute radiation pneumonitis (ARP) occurs within 1–3 months after radiotherapy. Typically, it is characterized by acute onset, high fever, choking cough with small mucous sputum amounts, chest pain, shortness of breath, and cyanosis, or even death due to respiratory distress syndrome or acute cor pulmonale [1]. After symptomatic disappearance in acute phase, gradually, ARP presents with chronic pulmonary fibrosis, chronic cor pulmonale, right heart insufficiency, respiratory failure, and other chronic complications, which affects the quality of life for patients and may be fatal [2]. Radiotherapy is one of the main therapeutic options for tumor patients. However, RP limits the radiation dose in thoracic tumor patients [3, 4]. About 50% of tumor patients in the United States choose and receive radiotherapy, with 70–90% of them being thoracic cancer patients [5]. Nuclear radiation accidents and pretreatment with bone marrow transplants have also been associated with RP occurrence. Thus, RP is a major clinical concern.

Conventional treatment options include antibiotics along with adrenocorticosteroids. Patients with severe dyspnea need continuous oxygen therapy or assisted ventilation. These therapeutic measures can temporarily relieve disease symptoms; however, their long-term efficacy is not sufficient. Moreover, the adverse effects of hormones, such as hormone-mediated immune inhibition, induction of double infections, and osteoporosis, limit their clinical applications.

Various drugs with reactive oxygen scavenging effects, such as sulfhydryl compounds, antioxidant enzymes and their analogues, and plant antioxidants [6, 7], can be used as radioprotective agents to remove free radicals produced by radiation or other cytotoxic drugs in normal tissues. However, these treatment forms are costly and may cause serious toxic side effects. The pathogenesis of RP has been well elucidated, but, currently, there are no effective preventive and treatment options for the disease. Therefore, it is vital to find targets or drugs with protective effects on normal lung tissues to reduce radiotherapy-associated lung injuries in lung cancer patients [8]. Traditional Chinese medicine (TCM) has great significance in preventing and improving the clinical symptoms and severity of RP. Furthermore, TCM can act on multiple targets and offer a comprehensive advantage for diseases with complex pathogenesis.

**Figure 1:**
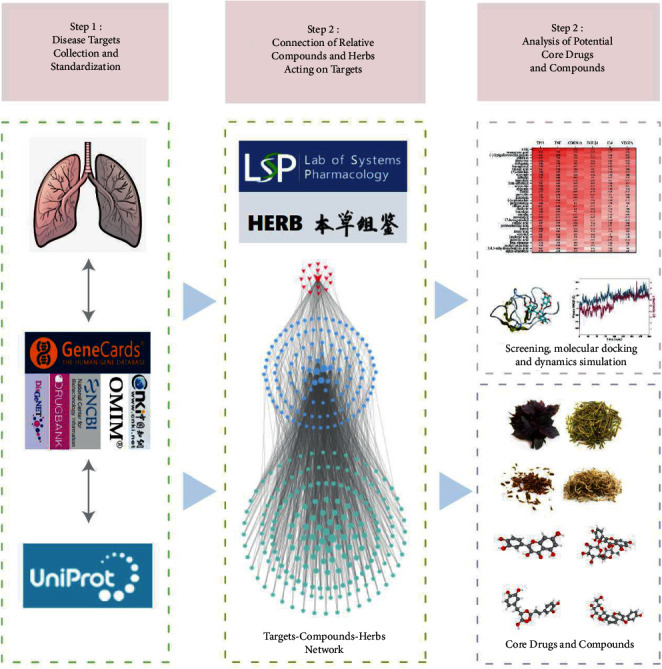
The whole framework based on network pharmacology.

In this study, through the network pharmacology approach, the RP-related targets were taken as entry points and matched to their corresponding ligand compounds in Chinese medicine. We aimed at investigating the complex connection between targets, compounds, and Chinese medicine. Moreover, we assessed the mechanisms of action and general rules of Chinese medicine in RP. Our findings provide a theoretical basis for subsequent exploration of Chinese medicine, therapeutic strategies, and clinical integration of Chinese medicine into Western medicine in RP management. A flow chart of this study is shown in Figure 1.

## 2. Materials and Methods

### 2.1. Collection of RP-Related Targets

The targets related to mechanisms of action of RP were obtained from the following databases: GeneCards (https://www.genecards.org/) [9], OMIM (https://omim.org) [10], TTD (therapeutic Target Database, bidd.nus.edu.sg) [11], DrugBank (https://www.drugbank.ca/) [12], and the Herb database (https://herb.ac.cn/) [13]. Targets identification was done using the key words; “radiation pneumonitistis”, “radiation pneumonia”, “acute radiation pneumonitis”, “radiation induced pneumonia”, “radioactive pneumonitis” and “radiation-induced pneumonitis”. The full names for each target were determined in UniProt protein database (https://www.uniprot.org/) [14].

### 2.2. Selection of Alternative Compounds and “Compound-Target” Network Construction

The Traditional Chinese Medicine Systems Pharmacology Database and Analysis Platform (TCMSP) (https://tcmspw.com/) [15] was used to obtain compounds that could act on RP-related targets.

Candidate compounds were selected based on the ADME index and oral bioavailability (OB) ≥30% and drug half-life (HL) >4 as well as drug likeness (DL) ≥0.18 [16]. After initial screening, deleted components were checked one by one after which relevant active ingredients were added. Given that composition information in the TCMSP database has computer prediction results, some of the data may not correspond to actual applications. Thus, screened compounds were used as candidate compounds, which were then imported into Cytoscape 3.7.2 software [17] along with targets to construct a “target-compound” network. Thereafter, topological properties of the network nodes were analyzed.

### 2.3. Herbal Acquisition and “Target-Compound-Chinese Medicine” Network Construction

The TCMSP database was used to acquire TCMs containing alternative compounds and to construct the alternative “compound-Chinese medicine” network. In conjunction with “compound-target” network, topological parameters for each node in the network were determined using the NetworkAnalyzer module of Cytoscape 3.7.2 to identify key nodes. Then, core drugs were selected to construct the “target-compound-Chinese medicine” network in order to comprehensively evaluate the efficacy of different compounds and Chinese medicine in RP treatment.

### 2.4. Molecular Docking Assessment

High ranking target proteins were screened by Cytoscape “target compound network”. Then, 3D crystal structures of target proteins were downloaded from the RCSB database (https://www.rcsb.org/pdb) and converted into PDB files for storage using Open Babel 3.1.1 (https://openbabel.org/wiki/Main_Page) [18]. Then, AutoDock Tools 1.5.6 (https://autodock.scripps.edu/resources/tools) were adopted for dehydrating and hydrogenation, calculating the Gasteiger charge, and storing it as a PDBQT file [19]. The potential core distributer was minimized by energy processing and the ligand atom type assigned to calculate the charge, which was stored in a PDBQT format. Molecular docking and docking scoring were performed using Moe2019 [20]. Active ingredients and targets with good binding activities were screened by Affinity. Docking score ≤−4.25 kcal/mol implies binding activities between the ligand and target and score ＜−5.0 kcal/mol indicates a good binding activity, while score ≤−7.0 kcal/mol implies strong docking activities [21].

### 2.5. Molecular Dynamics Simulation

MD simulations of docked complexes were performed using Desmond version 2020. The OPLS3e force field was used to initiate MD simulation, after which the system was solvated using a TIP3 water model. On postpreparation of the system, the production run was performed for 200 ns with a time step of 1.2 fs. Trajectory recording was done for every 200 ps, summing up to a recording of 10,00 frames. The root mean square deviation (RMSD) was calculated for backbone atoms and graphically analyzed to understand protein-ligand interactions. Root Mean Square Fluctuation (RMSF) for every residue was calculated to understand the major conformational changes in residues between the initial and dynamic state.

## 3. Results

### 3.1. Results of Target Acquisition

In this study, 96, 7, 2, and 0 RP-related targets were, respectively, obtained from the GeneCards, OMIM, TTD, and DrugBank databases. The targets were complemented with the Herb database, and duplicate values were removed after which the targets were matched with the TCMSP database. Unmatched targets were eliminated. Finally, 55 related targets were obtained and standardized in the UniProt database.

### 3.2. Candidate Compounds and the Target-Compound Network

**Figure 2:**
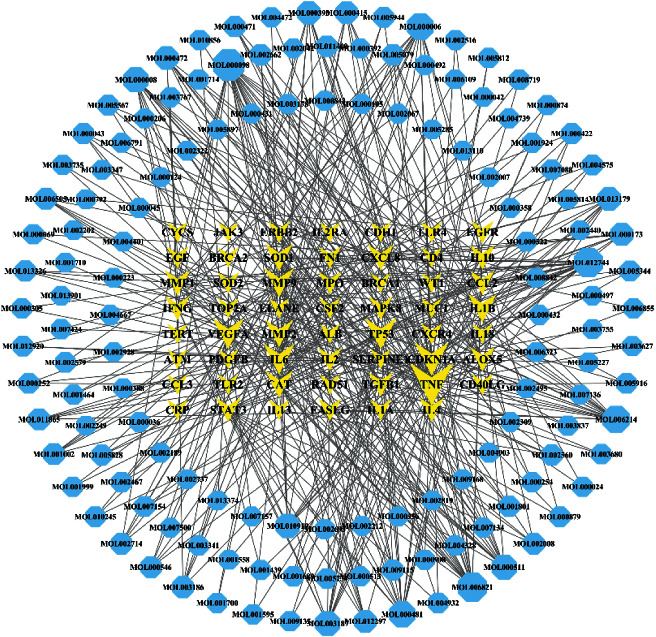
The target-compound network.

Fifty-five potential targets were matched with 12,144 small molecule compounds from the TCMSP database and a total of 137 potential compounds obtained.  Figure 2 shows the target-compound network constructed using potential targets and candidate compounds. The network has 106 nodes and 184 edges. The regular hexagon nodes represent candidate compounds while V-shaped nodes denote potential targets. Edges indicate the relationship between nodes while degree values show the number of edges connected to the node. Moreover, the size of the graph indicates degree values of the nodes. The larger the graphs, the more significant the influence in the network. Compounds with the top 10-degree values were quercetin, resveratrol, (-)-epigallocatechin-3-gallate (EGCG), progesterone, luteolin, genistein, apigenin, ursolic acid, triptolide, and 17-beta-estradiol. These compounds may interact with related target proteins in RP with high activity. The top 10 target proteins included Tumor Necrosis Factor (TNF), Cyclin Dependent Kinase Inhibitor 1A (CDKN1A), Tumor Protein 53 (TP53), Vascular Endothelial Growth Factor A (VEGFA), Interleukin-6 (IL-6), Transforming Growth Factor-*β* (TGF-*β*1), Matrix Metalloproteinases 9 (MMP9), Interleukin-1*β* (IL-1*β*), Catalase (CAT), and Matrix Metalloproteinases 2 (MMP2). These findings suggest that these target proteins are involved in RP treatment.

### 3.3. Candidate Compounds and Target-Compound Network

Based on the 137 candidate compounds, screening of TCMSP and chemical databases revealed 469 Chinese herbs. The compound-Chinese medicine network was constructed based on the relationship between compounds and traditional Chinese medicine. The network had 605 nodes and 2625 edges. Median degree values in the network were calculated twice and a value of 20 is obtained. A degree value >50 indicates a potential core compound. Sixteen potential core compounds were identified in the network.  Table 1 shows the top 5 potential core compounds (beta-sitosterol, palmitic acid, quercetin, kaempferol, and luteolin) ranked according to their degree values. Degree, betweenness centrality, and closeness centrality of potential core compounds were calculated using Network Analyzer in Cytoscape 3.7.2. The degree, betweenness centrality, and closeness centrality of beta-sitosterol were 247, 0.269, and 0.493, respectively. Beta-sitosterol was predicted to be the main compound that can be used to intervene RP because its topological parameters had the highest value. Palmitic acid ranked second with a degree, betweenness centrality, and closeness centrality of 239, 0.274, and 0.487, respectively. Quercetin ranked third with a degree, betweenness centrality, and closeness centrality of 289, 0.139, and 0.445, respectively. The top 10 types of traditional Chinese medicines ranked according to their degree values were YinXingYe, YuanSui, GeHua, MaHuang, ZiSu, SangYe, HongHua, ShaJi, LianQiao, and JinYinHua. Thereafter, based on bridging level between compounds, traditional Chinese medicine targets that were related to RP treatment were identified. The top-ranking compounds were Zi Su, Ge Hua, Ma Huang, and Jin Yin Hua (Table 2). Thus, RP treatment might involve regulation of different targets and pathways by traditional Chinese medicine.

**Figure 3:**
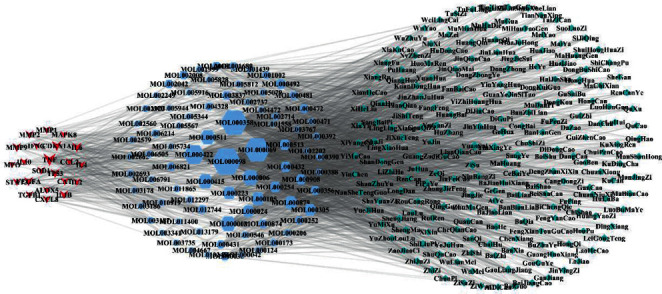
The target-compound-traditional Chinese medicine network (degree ≥5).

To understand the relationship between the targets, compounds, and TCM applied in the treatment of RP, a target-compound-traditional Chinese medicine network was generated (Figure 3). The degree values of objects included in the network were higher than 5. In Figure 3, the diamond, hexagon, and V shapes represent traditional Chinese medicine, compounds, and targets, respectively. In addition, the icon size was positively correlated with the degree values. Calculation of the median degree value of the compounds was performed thrice and a value of 41.5 was obtained. Core compounds were those with a degree value equal to or greater than 41.5. As a result, 37 compounds were identified as core compounds with the top 5 being beta-sitosterol, palmitic acid, quercetin, kaempferol, and luteolin.

### 3.4. Molecular Docking Results

The 37 potential core compounds were molecularly docked to six-core targets, namely, CDKN1A (PDBID: 6P8H), IL-6 (PDBID: 2ARW), TGFB1 (PDBID: 5VQP), TNF (PDBID: 2AZ5), TP53 (PDBID: 1JSP), and VEGFA (PDBID: 4KZN). Notably, 222 sets of receptor-ligand docking results were obtained. In the results, 208 sets had an affinity score <−5 kcal/mol, whereas 41 sets had an affinity score <−7 kcal/mol, culminating in a mean affinity of −6.17 kcal/mol. This suggested that the screened core compounds had a good binding affinity to the core targets.

**Figure 4:**
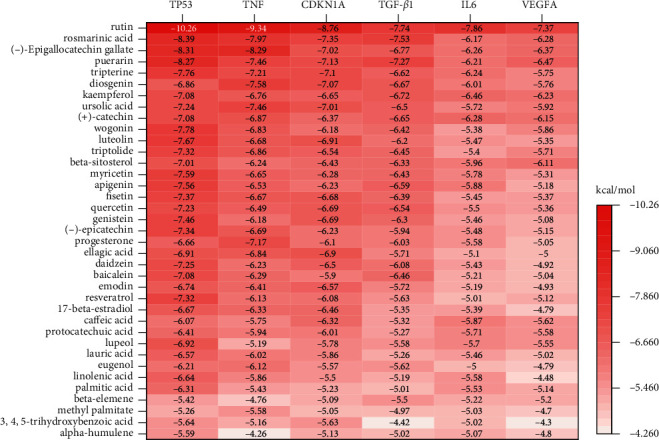
A heat map of molecular docking results between the core compounds and targets.

Out of the 222 identified groups, 95 were present in the target-compound network and were therefore referred to as the in-network combination. Notably, TNF-Rutin had the highest molecular docking score (−9.34 kcal/mol), followed by TP53-EGCG (−8.31 kcal/mol), and then TNF-EGCG (−8.29 kcal/mol). On the other hand, TNF-Alpha-humulene had the lowest molecular docking score (−4.26 kcal/mol). The average affinity score of the above combinations was −6.34 kcal/mol, indicating high interaction between compounds and targets in the target-compound network. Additionally, 127 new combinations were discovered outside the network, among which TP53-Rutin had the highest affinity score (−10.26 kcal/mol), followed by CDKN1A-Rutin (−8.76 kcal/mol) and TP53-rosmarinic acid (−8.39 kcal/mol). Further, VEGFA-3,4,5-trihydroxybenzoic acid had the lowest score (−4.3 kcal/mol) with a mean affinity score of −6.05 kcal/mol. These results demonstrated active binding between the core compounds and core targets.  Figure 4 shows that Rutin, rosmarinic acid, (-)-epigallocatechin-3-gallate, and puerarin showed strong binding to other core targets.

**Figure 5:**
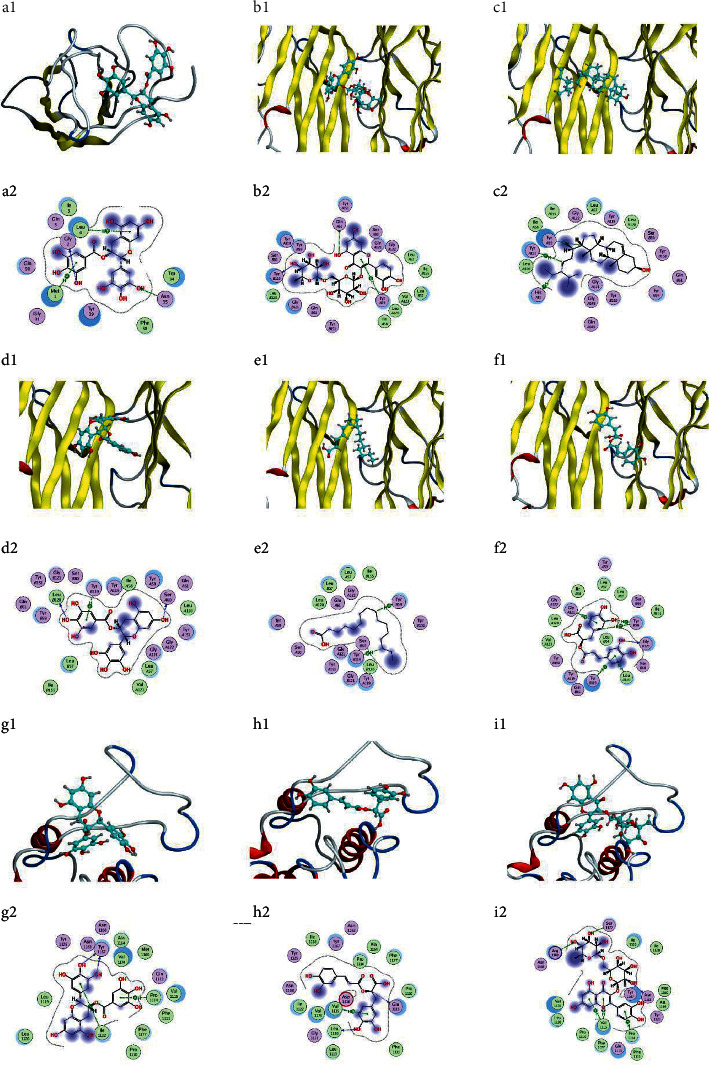
Molecular docking models. (a) IL-6-EGCG, (b) TNF-Rutin, (c) TNF-*β*-sitosterol, (d) TNF-EGCG, (e) TNF-palmitic acid, (f) TNF-rosmarinic acid, (g) TP53-EGCG, (h) TP53-rosmarinic acid, and (i) TP53-Rutin.Notes: in the 3D structure of ligand-protein complexes, the protein skeleton is represented with a tube shape, different peptide chains are given different colors, and ligands are represented with a blue bar. In the 2D interaction model, the protein residues are colored in circles based on their properties, green: hydrophobicity residues; purple: polarity residues.

In addition, nine ideal combinations were identified in the molecular docking experiments based on the affinity scores and the degree values of the target-compound-traditional Chinese medicine network. The three-dimensional and two-dimensional molecular docking models are shown in Figure 5. The models show that the ligands were embedded in the active pockets of the targets. In addition, the ligands interacted with multiple residues on the targets through hydrophobic interactions and hydrogen bonds.

### 3.5. Molecular Dynamics Simulation

**Figure 6:**
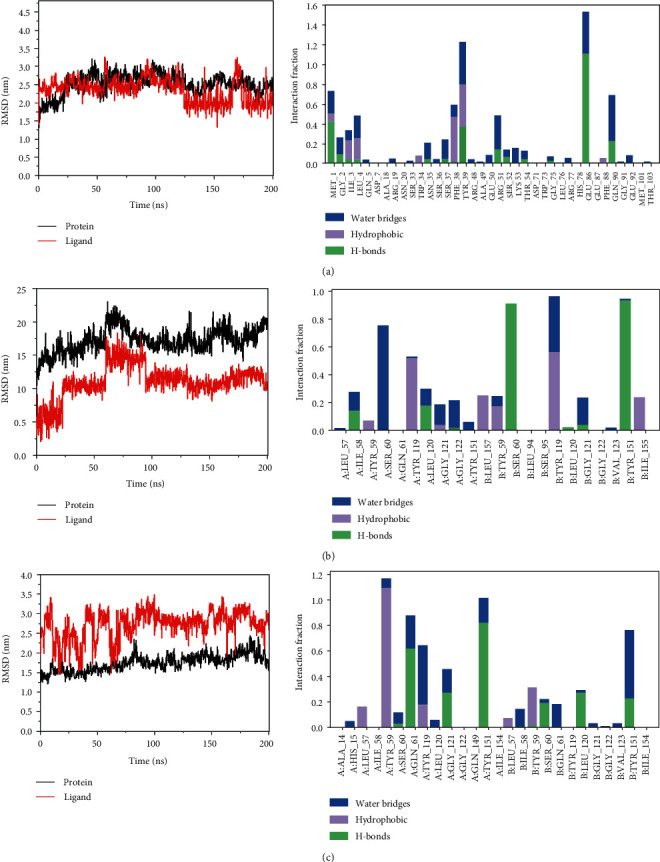
RMSD plot showing the molecular dynamics simulations. (a) The RMSD of IL-6 with Rutin. (b) The RMSD of TNF with epigallocatechin. (c) The RMSD of TNF with Rutin (black polygonal line means protein; red polygonal line means ligand).

The protein alpha root mean square deviation (RMSD) data of Rutin and IL-6 and epigallocatechin and TNF as well as Rutin and TNF are presented in terms of proteins and ligand as shown in Figures 6(a)–6(c).  Figure 6(a) shows that the composite protein of Rutin and IL-6 became stable after 25 ns, with a deviation of 2.5. Moreover, Figures 6(b) and 6(c) indicate that the system of TNF and epigallocatechin reached equilibrium at 60 ns. The system of TNF and Rutin reached equilibrium at 75 ns. These findings show that a stable conformation was achieved during MD simulation.

### 3.6. Statistics and Frequency Analysis of Flavour, Nature, and Meridian

**Figure 7:**
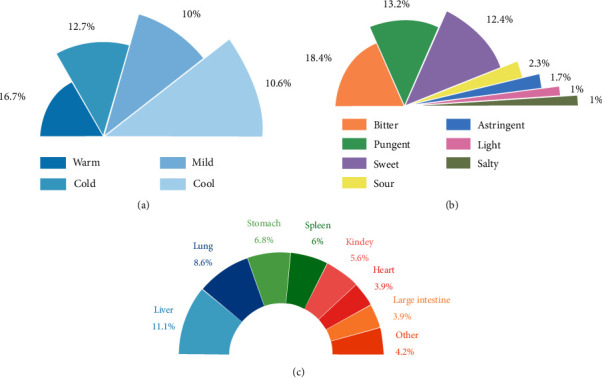
Information on Chinese medicine ((a) nature; (b) flavour; (c) meridian).

Information on flavour, nature, and meridian was collected from 469 traditional Chinese herbs. The results of frequency statistical analysis conducted using Excel program showed that, in terms of nature, bitter (225), acrid (183), and sweet (174) had the highest ranking. On the other hand, the proportion of warm (25.6%) and cold (25.2%) herbs was the highest. In addition, the highest ranked meridians were liver (17.1%), stomach (13.6%), and spleen (11.9%), which were involved in the treatment of RP. The results of frequency statistical analysis are shown in Figure 7.

## 4. Discussion

Radiation pneumonitis is a radiotherapy-associated complication during malignant tumor treatment. However, RP is not found in the well-known TCM literature, even though according to its symptoms, it belongs to the “cough,” “lung carbuncle,” “atrophic lung disease,” and “Pulmonary palsy” categories. Clinically, radiation is believed to be the main source of heat toxicity. Lungs are too delicate for excess cold and heat. During radiotherapy, heat toxicity accumulates in the lungs, leading to considerable organ damage. A combination of deficiency-heat and blood stasis can further destroy the lungs, resulting in the loss of normal lung functions. The tumor itself can destroy the body and heat-toxicity invades body, leading to qi collapse, which results in atrophic lung disease. Therefore, heat toxicity, deficiency of Yin, and static blood are the main pathological features of RP. Lung disease pathogenesis involves blood stasis as well as the deficiency of both qi and yi. The main treatment modes involve heat clearance, toxin removal, nourishing qi and yin, activating blood, and resolving stasis [22]. However, often, different doctors have distinct views. For instance, Professor Wang suggests that although radiation pneumonia has such pathological features as heat toxicity, lung dryness, yin and qi deficiency as well as fluid deficiency, it is caused by a wind pathogen, it occurs in the lungs, the primary cause is liver, insufficient lung fails to restrict liver are considered to be one of the pathogenesises. Therefore, the main treatment forms involve dispersing wind and relieving cough [23]. Besides, Professor Shu found that RP is more likely to occur during seasonal changes or in the influenza period and is usually caused by respiratory tract infections. Therefore, Professor Shu cleared the heat and removed toxins when treating patients who had received chest radiotherapy. In addition, he dispersed the wind and cooled the blood to prevent the disease [24].

We found that bitter, sweet, and spicy traditional Chinese herbs can potentially treat RP. A combination of cold and warm medicines may be more effective. The main channels belong to the liver meridian and lung meridian. These results correspond to clinical cognitive of lung injury caused by radiation therapy. Medicines that are warm or cold and spicy, sweet, or bitter have been reported to be more effective in clearing heat and removing toxins, nourishing qi and Yin, activating blood and resolving stasis, clearing heat, and promoting fluid production [25]. The bitter taste can resolve dampness and preserve yin and is often used to manage heat syndrome in TCM. Furthermore, acrid can be divergent, promoting the flow of qi and blood circulation. Additionally, it can diverge Yu fire and can also work with the bitter cold medicine to prevent fire. Sweet drugs can be tonic, slow, urgent and moderate. After radiotherapy, the human body consumes more energy, which manifests as deficiency of both qi and yin. Sweet and cool drugs can nourish qi and yin. Based on the four properties, including cool, cold, warm, and hot, most of the traditional Chinese medicines are warm and cold. Cold herbs clear heat, reduce fire, cool the blood, and remove toxins. A combination of bitter and cold herbal medicines clears heat and resolves dampness while a combination of sweet and cold medicines nourishes yin and clears heat, consistent with pathogenesis of RP lung heat and yin injury. According to the meridian tropism theory, RP pathogenesis involves wood depression transforming into wind and wood fire tormenting metal. Wind medicine can enter the liver meridian and help to grow upwards. Notably, leaves of ephedra and loquat can regulate dispersion in the lungs.

Modern medicine has not conclusively determined the mechanisms of RP, and a number of theories have been put forward. For instance, the cytokine theory suggests that RP occurs due to radiation-associated activations of macrophages, mast cells, lung interstitial cells, alveolar epithelial cells, and other target cells in the irradiation field. The activated cells release large amounts of proinflammatory cytokines that locally accumulate, thereby regulating and accelerating inflammatory responses. Cytokines that are closely related to RP occurrence and development include Transforming Growth Factor-*β*1 (TGF-*β*1), Tumor Necrosis Factor-*α* (TNF-*α*), Interleukin-6 (IL-6), and Platelet-Derived Growth Factor (PDGF). Notably, TGF-*β*1 is an important cytokine for predicting RP occurrence. Radiation causes the TGF-*β*1 produced during inflammatory responses in lung tissues to activate the phosphatidylinositol 3-kinase-serine/threonine protein kinase (PI3K/AKT) signaling pathway, which promotes alveolar epithelial cell transformations into lung interstitial cells [26], further causing RILI. Elevated TGF-*β* levels can also be used to diagnose early-stage RP and late-stage radioactive pulmonary fibrosis. Moreover, IL-6 has dual effects of inducing and inhibiting inflammation. Zhang et al. reported that RP patients have elevated IL-6 levels, relative to those without the disease, suggesting that IL-6 is involved in RILI processes after radiotherapy [27]. A previous study reported a significant increase in IL-1*β* levels after radiation [28]. Biologically, TNF comprise a family of cytokines that can damage tumor cells and cause necrosis. Among the TNFs, TNF-*α* is an initiator of the cytokine regulatory network in the body [29]. TNF-*α* also plays an important role in inducing apoptosis, regulating immune responses, and initiating as well as maintaining the occurrence and development of RP. Rübe et al. [30] showed that the timepoint at which plasma TNF-*α* levels in lung cancer patients increased before and after radiotherapy coincided with the period during which RP occurred, indicating that, after irradiation, TNF-*α* is involved in inflammatory responses in the lungs.

In the target-compound network constructed in this study, the highest ranked targets included TP53, VEGFA, SOD, and CAT, in addition to TGF-*β*1, IL-6, and TNF. It was also predicted that these targets are important in regulating RP. TP53 is a tumor suppressor gene that is expressed in low levels in normal cells, but highly expressed in malignant tumors. VEGFA, a member of the Platelet-Derived Growth Factor (PDGF)/Vascular Endothelial Growth Factor (VEGF) family, is the main inducer of angiogenesis. In addition, PDGF promotes pulmonary vascular smooth muscle cell proliferation and angiogenesis. Radiotherapy-induced liver fibrosis is often accompanied by angiogenesis [31], suggesting that VEGFA may play a significant role in RP. Oxidative stress is one of the mechanisms involved in RILI pathogenesis [32]. Therefore, it is possible that radiation-induced cytotoxicity is mediated by Reactive Oxygen Species (ROS). Notably, SOD and CAT constitute the body's first line of defense against reactive oxygen species. Additionally, given that its overexpression significantly reduced inflammation in the lungs and oxidative damage in RILI mice models, SOD has some anti-inflammatory effects and can inhibit lung fibrosis [33].

Target-compound screening revealed that quercetin, resveratrol, and epigallocatechin gallate (EGCG) had a higher connectivity degree. Quercetin, resveratrol, and EGCG have potent antioxidant, anti-inflammatory, and antitumor activities [34–36]. Quercetin is a polyphenolic flavonoid compound that can significantly reduce inflammatory exudation of radiation-induced lung injury and has a certain protective effect against RP [37]. Resveratrol, a natural polyphenol that occurs as a phytoalexin, has certain protective effects against RP and enhances the radiosensitivity of lung cancer cells [38, 39], which may be related to its ability to suppress TGF-*β*1 levels. EGCG, the major polyphenolic component of dried green tea extracts, can reduce serum levels of inflammatory cytokines, IL-6, IL-10, and TNF-*α* in lung injury. In radiation-induced lung injury, EGCG suppresses early inflammatory responses and late pulmonary fibrosis [40]. Moreover, EGCG can reduce ionizing radiation-mediated damage by scavenging for free radicals, resisting oxidative stress, and hindering apoptosis [41–43]. The compound-Chinese herb network in this study showed that palmitic acid and kaempferol are important compounds that can interfere with RP progression through multiple targets. However, it has not been established whether they can affect RP development.

Based on degree value and number of targets, the most important traditional Chinese herbs were *Perilla pueraria*, ephedra, honeysuckle, sea buckthorn, *Ginkgo biloba,* and cuspidatum. Pueraria contains flavonoid components such as quercetin and genistein. Previous studies reported a significant increase in SOD and CAT activities in high dose Pueraria polysaccharide group after irradiation of mice with X-rays, implying that the Pueraria polysaccharide has a certain preventive and protective effect against radiation-associated damage [44]. Biologically, TNF-*α* is a negative regulator of hematopoiesis. Treatment of radiation-injured mice with flavonoids from the sea buckthorn suppressed TNF-*α* levels [45, 46]. TNF-*α* can also suppress radiation-induced bone marrow cell apoptosis, which provides a basis for applications of traditional Mongolian medicine, Hippophae, in radiation damage. Moreover, the *Ginkgo biloba* extract increased the number of lymphocytes in irradiated mice, mitigated radiation-associated damage to the liver synthesis function, increased SOD as well as GSH activities, and suppressed malondialdehyde (MDA) levels, a product of membrane lipid peroxidation. Honeysuckle and *Polygonum cuspidatum* have the functions of clearing heat and removing toxicity [47]. Resveratrol from *Polygonum cuspidatum* was shown to inhibit radiation-induced apoptosis of spleen cells in mice [47]. Additionally, a high dose of the ephedra compound suppressed VEGF levels in tumor tissues of lung cancer mice models, thereby inhibiting tumor angiogenesis [49]. Therefore, ephedra has the potential for RP prevention and treatment. Studies on prevention and treatment of RP using Perilla or on the role of Perilla in radiation resistance are few. However, its targets have many intersections with RP disease targets, suggesting that it is a promising drug candidate for RP prevention and treatment. Overall, most of the important traditional Chinese herbs in the network diagram are used for relieving exterior disorders. Therefore, RP treatment should focus on early exiting of evil, guiding it from its entry to its exit, and pay attention to the existence of exterior syndromes of TCM. Moreover, it is possible to prevent and cure RP using ROS-targeted treatments, inhibiting the release of inflammatory factors and activation of related signaling pathways.

Molecular docking showed that Rutin, rosmarinate, and Puerarin had good molecular docking scores. Rutin regulates the immune system by inhibiting radiation-induced oxidative and inflammatory stress, which helps in minimizing radiation-induced pneumonia and fibrosis [48]. Rosmarinic acid suppresses intracellular production of ROS, VEGF, and IL-8 levels [49]. Rosmarinic acid may also play a role in interfering with RP progression. Puerarin exhibited protective effects in experimental rats with ionizing radiation injury [50]. The study also showed that, after exposure to radiation, Puerarin can effectively resolve the decrease in red and white blood cells in peripheral blood of rats. Puerarin-associated protective effects may be related to elevated SOD activities and suppressed MDA levels. These findings imply that a large number of active components in Chinese medicines can interfere with RP occurrence and development. However, the associated mechanisms of action have not been elucidated. Molecular dynamics simulation results showed that both compounds could stably bind the receptor, proving the reliability of the predicted efficacy results.

Based on the network pharmacology method, we established an RP-related target-compound-Chinese medicine network. In addition, the potential Chinese herbs that may interfere with the RP process and the possible molecular basis as well as mechanisms of action were explored. The 55 RP targets were matched to 137 candidate compounds and 469 Chinese herbs. The most important Chinese herbs were identified to be Perilla, Pueraria, ephedra, honeysuckle, sea buckthorn, *Ginkgo biloba*, and cuspidatum, highlighting some new drug candidates for RP treatment. Nonetheless, their mechanism of actions should be explored further. Despite the insightful findings, this study has some limitations. For example, due to incomplete information in the database, some active ingredients of core targets may have been omitted during the screening process. Therefore, the scope of database screening should be expanded to explore the role of important target components in improving the pathological processes of RP. This will help in identification of more comprehensive drug candidates and better explain the mechanisms of drug intervention in RP.

## Figures and Tables

**Table 1:** The top 5 potential core compounds ranked according to the degree value.

Mol ID	CAS	Mol name	Degree	Betweenness centrality	Closeness centrality
MOL000358	83-46-5	Beta-sitosterol	247	0.268815	0.493
MOL000069	67701-02-4	Palmitic acid	239	0.274079	0.487
MOL000098	73123-10-1	Quercetin	189	0.13975	0.444
MOL000422	520-18-3	Kaempferol	134	0.074748	0.407
MOL000006	491-70-3	Luteolin	93	0.045981	0.379

**Table 2:** The major traditional Chinese medicines.

Traditional Chinese herbs	Degree	Traditional Chinese herbs	Number of targets
Yin Xing Ye	23	Zi Su	41
Yuan Sui	22	Ge Hua	39
Ge Hua	21	Ma Huang	39
Ma Huang	21	Jin Yin Hua	38
Zi Su	20	Pa Pi Ye	38
Sang Ye	19	Hu Zhang	37
Hong Hua	18	Nu Zhen Zi	37
Sha Ji	17	Sha Ji	37
Lian Qiao	17	Xiang Ru	37
Jin Yin Hua	16	Yin Xing Ye	37

## Data Availability

The datasets used and/or analyzed in the current study are available from the corresponding author upon reasonable request.

## Abbreviations

TCM: Traditional Chinese medicine
TCMSP: Traditional Chinese Medicine Systems Pharmacology
RP: Radiation pneumonitis
RILI: Radiation-induced lung injury
ARP: Acute radiation pneumonitis
DL: Drug likeness
OB: Oral bioavailability
HL: Half-life
TTD: Therapeutic Target Database
EGCG: (-)-Epigallocatechin-3-gallate
TNF: Tumor necrosis factor
CDKN1A: Cyclin Dependent Kinase Inhibitor 1A
TP53: Tumor protein 53
VEGFA: Vascular endothelial growth factor A
TGF-*β*1: Transforming growth factor-*β*
MMP9: Matrix Metalloproteinases 9
IL-1*β*: Interleukin-1*β*
CAT: Catalase
MMP2: Matrix Metalloproteinases 2
SOD: Superoxide dismutase
TNF-*α:*: Tumor Necrosis Factor-*α*
IL-6: Interleukin-6
PDGF: Platelet-Derived Growth Factor
PI3K/AKT: Phosphatidylinositol 3-kinase-serine/threonine protein kinase
VEGF: Vascular endothelial growth factor
ROS: Reactive oxygen species
MDA: Malondialdehyde.
